# NGS-Based Genomic Characterization of ESBL/AmpC-Producing Extraintestinal Pathogenic *Escherichia coli* from Captive Wildlife in Tunisia

**DOI:** 10.3390/antibiotics15050449

**Published:** 2026-04-29

**Authors:** Zaineb Hamzaoui, Hajer Kilani, Sana Ferjani, Elaa Maamar, Ahmed Fakhfakh, Lamia Kanzari, Ilhem Boutiba-Ben Boubaker

**Affiliations:** 1 LR99ES09, Faculty of Medicine of Tunis, University of Tunis El Manar, Tunis 1007, Tunisia; hajerkilani@yahoo.fr (H.K.); sana.ferjani@rns.tn (S.F.); elaa.maamar@fst.utm.tn (E.M.); ahmed81fakhfakh@gmail.com (A.F.); lamia.kanzari@fmt.utm.tn (L.K.); ilhem.boutiba@rns.tn (I.B.-B.B.); 2 Laboratory of Microbiology, Charles Nicolle Hospital, Boulevard 9 Avril, Tunis 1006, Tunisia

**Keywords:** *Escherichia coli*, extraintestinal pathogenic *E. coli*, ESBL, AmpC, multidrug resistance, captive wildlife, One Health, whole-genome sequencing, ST69, ST162

## Abstract

**Background/Objectives:** Multidrug-resistant (MDR) *Escherichia coli* resistant to third-generation cephalosporins are a growing One Health concern, but data on extraintestinal pathogenic *E. coli* (ExPEC) from wildlife in North Africa remain scarce. We aimed to characterize ESBL/AmpC-producing ExPEC from captive wild mammals in Tunisia and to situate these isolates in a global genomic context. **Methods:** In 2018, 30 fecal samples from 14 captive wild mammals in a private farm were screened on cefotaxime agar. Four cefotaxime-resistant *E. coli* isolates were recovered from a llama, lion, hyena, and tiger. Antimicrobial susceptibility testing and Illumina whole-genome sequencing were combined with in silico typing, resistome and virulome profiling, plasmid and mobile element analysis, human pathogenicity prediction and core-genome MLST-based minimum-spanning trees. **Results:** All isolates were MDR but remained susceptible to carbapenems, colistin and tigecycline. Two ST162/B1 isolates from the llama and tiger carried *bla*_CMY-2_, whereas two ST69/D isolates from the lion and hyena harbored *bla*_CTX-M-15_ and *qnrS1*. Genomes encoded 61–68 antimicrobial resistance genes and 114–131 virulence-associated genes, together with IncF-, IncI1- and IncY-type plasmids and IS26-rich insertion sequence profiles. Mating-out assays yielded cefotaxime-resistant transconjugants, supporting plasmid transferability of *bla*_CMY-2_ or *bla*_CTX-M-15_. PathogenFinder predicted a ≥0.93 probability of human pathogenicity for all isolates. cgMLST-based trees showed that Tunisian ST69 and ST162 clustered within internationally disseminated lineages containing human, animal and food isolates, rather than forming wildlife-restricted branches. **Conclusions:** Captive wild mammals in Tunisia can harbor high-risk ExPEC lineages combining ESBL/AmpC production, multidrug resistance and extensive virulence and mobility gene repertoires. These findings highlight captive wildlife as potential reservoirs and sentinels of clinically relevant *E. coli* and underscore the need for integrated WGS-based One Health surveillance at the human–animal–environment interface in North Africa.

## 1. Introduction

Antimicrobial resistance (AMR) in Gram-negative bacteria is now recognized as a major threat to human and animal health worldwide, compromising the efficacy of critically important antibiotics and increasing the burden of difficult-to-treat infections [1]. Among these pathogens, *Escherichia coli* plays a central role as both a commensal inhabitant of the intestinal microbiota and an opportunistic pathogen responsible for a wide range of extra-intestinal infections in humans and animals, including urinary tract infections, sepsis, and meningitis [2]. The emergence and dissemination of third-generation cephalosporin-resistant *E. coli*, frequently mediated by extended-spectrum β-lactamases (ESBLs) or plasmid-encoded AmpC enzymes, are of particular concern in clinical and community settings [3].

The ecology of AMR is complex and aligns closely with the One Health concept, which emphasizes the interconnectedness of human, animal, and environmental health. Resistant *E. coli* can circulate between humans, livestock, companion animals, and wildlife, facilitated by direct contact, contaminated food chains, shared water sources, and environmental reservoirs [4]. Wild animals, including those living in proximity to human activities or kept in captivity, may act as sentinels or reservoirs for multidrug-resistant (MDR) *E. coli* and other Enterobacterales, reflecting local antibiotic use and contamination pressure in surrounding ecosystems [5]. However, the contribution of wildlife to the broader AMR landscape remains incompletely characterized, especially in low- and middle-income countries.

Captive wild mammals in farms, zoos, and wildlife parks occupy a unique ecological niche at the interface between the environment, domestic animals and humans. They can acquire MDR bacteria from contaminated feed, water, soil, fomites, caretakers or nearby livestock, and may in turn shed these organisms back into the environment. Nevertheless, there are still relatively few whole-genome sequencing (WGS) studies focusing on the resistome, virulome and mobile genetic elements of MDR *E. coli* from wildlife, particularly in North Africa and the Mediterranean region.

Tunisia has documented a high burden of ESBL-producing *Enterobacterales* in hospitals [6] and, to a lesser extent, in community and livestock settings [7,8], but data on AMR in wildlife are scarce.

Understanding possible cross-species transmission pathways and evaluating the pathogenic potential of multidrug-resistant *E. coli* prevalent in captive wild animals is crucial in the context of the One Health initiative.

Here, we combined phenotypic antimicrobial susceptibility testing with short-read WGS to investigate four third-generation cephalosporin-resistant *E. coli* isolates recovered from fecal samples of a llama, lion, hyena, and tiger housed in a private wildlife farm in northeastern Tunisia.

To identify the genomic characteristics of these wildlife-associated ESBL/AmpC-producing *E. coli* and to situate these isolates within the global phylogenetic context using cgMLST-based minimum-spanning trees, we employed a thorough in silico pipeline that integrated resistome and virulome profiling, plasmid and mobile genetic element analysis, CRISPR–Cas, and in silico pathogenicity prediction.

## 2. Results

### 2.1. Sample Screening and Isolate Recovery

In 2018, a total of 30 fecal samples collected from 14 captive wild mammals were screened on cefotaxime-supplemented medium for third-generation cephalosporin-resistant Enterobacterales. Four cefotaxime-resistant *E. coli* isolates were recovered from four different host species: llama (Ec1), lion (Ec2), hyena (Ec3), and tiger (Ec4), corresponding to 4/30 (13.3%) screened samples and 4/14 (28.6%) animals sampled.

### 2.2. Phenotypic Antimicrobial Susceptibility Profiles

All four isolates displayed a multidrug-resistant (MDR) phenotype and were resistant to third-generation cephalosporins, consistent with the initial cefotaxime-based screening. Resistance was consistently observed against penicillins (ampicillin, ticarcillin), cephalosporins (including first-, third- and fourth-generation agents), aztreonam, tobramycin, nalidixic acid and fluoroquinolones (norfloxacin, ciprofloxacin), tetracycline, chloramphenicol, and trimethoprim–sulfamethoxazole. In contrast, all isolates remained susceptible to carbapenems (ertapenem, imipenem, meropenem), amikacin, colistin, and tigecycline (Table 1). MICs were determined for selected antibiotics (including colistin by broth microdilution and tigecycline by Etest) and were concordant with disk-diffusion results (Table 1; Supplementary Table S1). Phenotypic confirmatory testing supported an ESBL phenotype for Ec2 and Ec3 (positive DDST with clavulanate), whereas Ec1 and Ec4 were DDST-negative and showed an AmpC phenotype; the DDST repeated on Mueller–Hinton agar supplemented with cloxacillin (75 mg/L) yielded concordant results.

### 2.3. Genome Sequencing, Assembly Quality, and Global Annotation Metrics

Illumina short-read sequencing and de novo assembly produced high-quality draft genomes for the four isolates (Ec1–Ec4). Genome sizes ranged from 4.92 to 5.03 Mb with GC contents between 50.55% and 50.64%. Assemblies comprised 68–97 contigs with N50 values of 180,087–251,976 bp and L50 values of 8–9 (Table 2). RASTtk annotation predicted 3–5 rRNA operons, 75–80 tRNA genes, and 4735–5016 coding sequences per genome. Each genome harbored 61–68 antimicrobial resistance genes (CARD), 114–131 virulence-associated genes (VFDB), 142–146 metal-resistance genes (BacMet), and 942–977 predicted transporters (TCDB) (Table 2). Raw sequence reads for all four isolates have been deposited in the NCBI Sequence Read Archive (SRA) under BioProject accession PRJNA1367915.

### 2.4. Phylogenetic Background and in Silico Typing

In silico typing revealed two distinct clonal backgrounds among the wild-animal isolates. The llama (Ec1) and tiger (Ec4) strains belonged to ST162, phylogroup B1, with serotype O134:H19, whereas the lion (Ec2) and hyena (Ec3) carried ST69, phylogroup D, serotype O15:H18. Ribosomal MLST assigned rST 1544 to the ST162 isolates and rST 2135 to the ST69 isolates (Table 3). 

FimTyper identified fimH32 in ST162 genomes (Ec1/Ec4) and fimH27 in ST69 genomes (Ec2/Ec3); in combination with fumC65 (ST162) and fumC35 (ST69), these profiles were consistent with ExPEC-associated lineages (Table 3).

Using cgMLSTFinder v1.2 (CGE; software v1.0.1; https://cge.food.dtu.dk/services/cgMLSTFinder/ (accessed on 17 November 2025)) with the 2513-locus *Escherichia*/*Shigella* scheme, reliable core-genome sequence types were obtained for two isolates: Ec1 (cgST 207265; 94.3% loci called) and Ec3 (cgST 134012; 95.3% loci called). For Ec2 and Ec4, cgMLST profiles could not be retrieved with the standalone cgMLSTFinder call (no loci passed the internal allele-calling thresholds on the draft assemblies). Therefore, cgMLST-based comparisons and minimum-spanning trees were generated using the PubMLST *Escherichia/Shigella* cgMLST scheme (2513 loci), which returned callable profiles for all four genomes.

Species identity as *E. coli* and the ESBL phenotype were independently confirmed from raw reads by KmerResistance/KmerFinder, which consistently identified *E. coli* reference genomes as the best species match and detected the same key β-lactamase genes as assembly-based tools.

CRISPRCasFinder tool in Proksee (v1.1.0; https://proksee.ca/tools/crispr_cas_finder (accessed on 24 November 2025)) detected a complete type I-E CRISPR–Cas locus with two large CRISPR arrays in Ec3 and Ec4, whereas Ec1 and Ec2 displayed only short orphan CRISPR candidates without *cas* genes.

### 2.5. Resistome and Chromosomal Mutations

The four genomes carried a diverse set of acquired antimicrobial resistance genes. All isolates harbored *bla*_TEM-1B_ together with the glycopeptide resistance gene vanG and multiple aminoglycoside-modifying enzymes, including *aadA5*, APH(6)-Id, and APH(3″)-Ib, as well as *sul2* and *dfrA14*/*dfrA17* genes mediating sulfonamide and trimethoprim resistance. The tetracycline resistance gene tet(B) was present in the ST162 isolates (Ec1 and Ec4) (Table 3).

Regarding β-lactam resistance, the ST162/B1 strains (Ec1, Ec4) harbored the plasmid-mediated AmpC CMY-2, whereas the ST69/D strains (Ec2, Ec3) carried the ESBL CTX-M-15. These enzymes co-occurred with TEM-1B and additional narrow-spectrum β-lactamases (EC-18/EC-8). The ST69 isolates also contained the plasmid-mediated quinolone resistance gene *qnrS1*, while fluoroquinolone resistance in the ST162 isolates was mainly associated with chromosomal mutations (Table 3).

Consistent with the multidrug-resistant phenotype, all genomes exhibited multiple point mutations in antibiotic targets and regulatory loci. Fluoroquinolone resistance–associated substitutions *gyrA* S83L/D87N and *parC* S80I were found in the ST162 isolates, whereas ST69 genomes lacked *parC* mutations but retained wild-type quinolone target alleles. All four isolates carried the GlpT E448K substitution linked to fosfomycin resistance and several amino acid changes in PBP3 (FtsI; D350N, S357N) and *cyaA* (S352T in ST69) (Table 3).

Overall, the genotypic determinants were concordant with the phenotypic susceptibility profiles (Table 1 and Table 3). Resistance to third-/fourth-generation cephalosporins and aztreonam was consistent with the presence of *bla*_CTX-M-15_ in Ec2/Ec3 and *bla*_CMY-2_ in Ec1/Ec4, together with *bla*_TEM-1B_. The quinolone/fluoroquinolone-resistant phenotype aligned with *qnrS1* in Ec2/Ec3 and with quinolone target mutations (*gyrA* S83L/D87N and *parC* S80I) in Ec1/Ec4. Trimethoprim–sulfamethoxazole resistance was supported by *sul2* and *dfr* genes, and aminoglycoside resistance patterns were consistent with aminoglycoside-modifying enzyme genes (e.g., *aadA5* and *aph* variants). The intermediate fosfomycin phenotype across isolates matched the shared GlpT E448K substitution. Finally, the retained susceptibility to carbapenems and colistin was consistent with the absence of detected carbapenemase genes and plasmid-mediated colistin resistance determinants (*mcr*), and no acquired high-level tigecycline resistance gene was identified.

Moreover, efflux and global regulator genes showed multiple variants, including alterations in AcrR and MarR within the AcrAB–TolC efflux system (Y137H and G103S) and mutations in oxidative stress regulators soxR and soxS (Table 3).

### 2.6. Virulence Gene Content, Plasmid Replicons and Mobile Genetic Elements

Virulence profiling revealed a rich repertoire of genes associated with adhesion, iron acquisition, serum resistance, and toxin production. All isolates carried type 1 fimbriae (fimH) and multiple additional adhesins, alongside genes for curli (csgA) and outer-membrane proteases (ompT). Iron acquisition systems included iroN, iucC/iutA, and sitA in ST162 isolates and fyuA and irp2 in the ST69 group. Several toxins or toxin-like factors were detected, such as astA and hlyE, together with complement and serum resistance factors iss, traT, and the tellurite resistance gene terC (Table 3).

PlasmidFinder identified multiple incompatibility groups. The ST162/B1 isolates (Ec1, Ec4) carried a complex plasmidome including IncFIB, IncFIC(FII), IncI1-I(Alpha), and small Col-type plasmids (ColpVC, Col(MG828)), whereas the ST69/D isolates (Ec2, Ec3) harbored IncY and Col(MG828) replicons.

Mobile genetic element analysis showed numerous insertion sequences and transposons, dominated by IS26, ISEc9, IS629, and related IS families, with additional elements such as ISKpn19, ISEc38, IS4, and ISSfl10 depending on the isolate.

### 2.7. Transfer of Resistance

Conjugation assays supported plasmid-mediated transfer of key β-lactam resistance determinants. Cefotaxime-resistant transconjugants were obtained for all four donors. For the two AmpC-producing isolates (Ec1 and Ec4), *bla*_CMY-2_ and *bla*_TEM-1B_ were detected in the corresponding transconjugants, which displayed co-transfer of selected non-β-lactam resistance phenotypes (notably tetracycline and chloramphenicol, and trimethoprim–sulfamethoxazole for Ec4). For the two ESBL-producing isolates (Ec2 and Ec3), *bla*_CTX-M-15_ and *bla*_TEM-1B_ were detected in transconjugants, along with co-transferred resistance to trimethoprim–sulfamethoxazole, tetracycline, chloramphenicol, and ciprofloxacin (Table 4).

### 2.8. Predicted Human Pathogenicity

PathogenFinder classified all four genomes as likely human pathogens, with predicted probabilities ranging from 0.928 to 0.935. Predictions were supported by 760–897 matched protein sequences covering ~16–20% of each genome and by a marked excess of pathogenic over non-pathogenic protein family matches.

### 2.9. Core-Genome Relatedness in a Global Context

**Figure 1:**
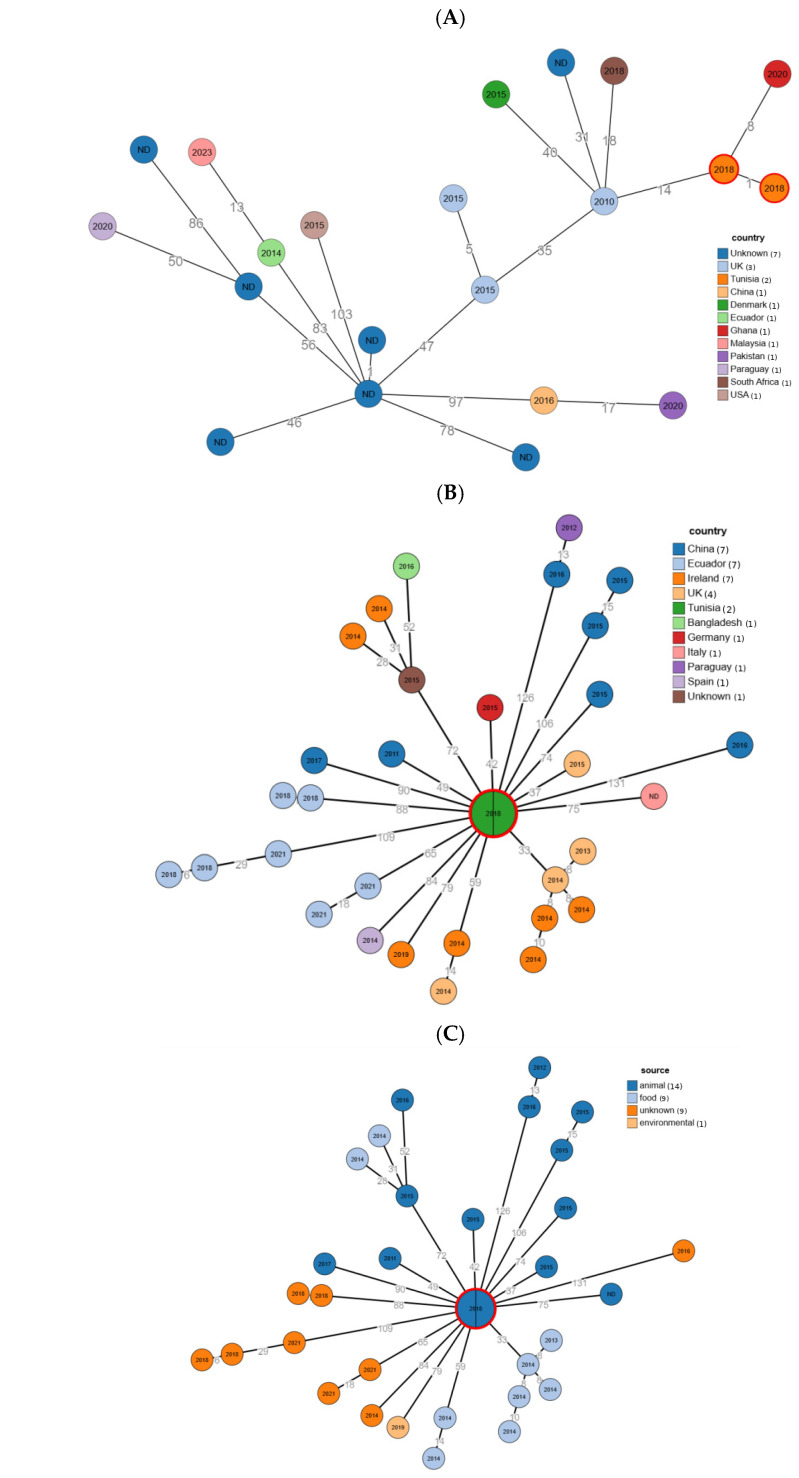

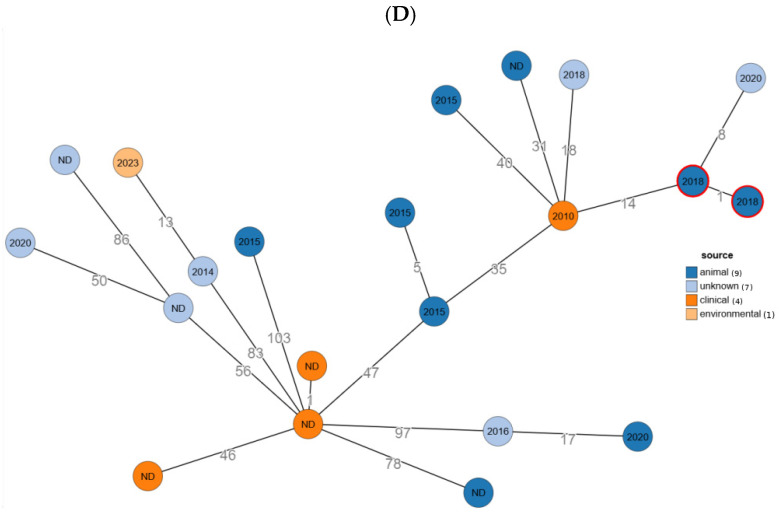
Core genome MLST-based minimum-spanning trees of *E. coli* ST69 and ST162 isolates from Tunisia and their closest relatives in the PubMLST database. (**A**) ST69 isolates colored according to country of isolation; (**B**) ST162 isolates colored according to country of isolation; (**C**) ST69 isolates colored according to source of isolation (animal, food, clinical, environmental, or unknown); (**D**) ST162 isolates colored according to source of isolation. Trees were generated from allelic profiles of 2513 core-genome loci using the cgMLST scheme implemented in PubMLST. Each node represents a unique allelic profile; node size is proportional to the number of identical isolates in the database. Tunisian isolates from this study (Ec1–Ec4) are highlighted/circled in red. Numbers on the branches indicate allelic differences between connected nodes, and numbers inside the nodes indicate the year of isolation (ND, no data).

Minimum-spanning trees based on the 2513-locus *Escherichia*/*Shigella* cgMLST scheme placed the Tunisian ST69 (Ec2, Ec3) and ST162 (Ec1, Ec4) isolates within internationally disseminated lineages rather than wildlife-restricted branches. Within ST69, Ec2 and Ec3 clustered tightly (1 allele difference) and fell within a sublineage including isolates from Ghana, the UK, South Africa, and Denmark; the closest neighbor was a Ghanaian isolate from 2020 (8 alleles), and a UK isolate from 2010 differed by 14 alleles (Figure 1A). Within ST162, Ec1 and Ec4 were indistinguishable by cgMLST and occupied a central position in a star-like cluster comprising isolates from multiple countries (e.g., UK, Germany, China, Ecuador, Italy), with distances to the closest PubMLST neighbors ranging from 33 to 131 alleles (Figure 1B). When mapped by source, the ST162 comparison set included 14/33 animal and 9/33 food isolates (with 9/33 unknown and 1/33 environmental), whereas the ST69 set included 9/21 animal and 4/21 clinical isolates (with 7/21 unknown and 1/21 environmental) (Figure 1C,D).

## 3. Discussion

The recovery of four third-generation cephalosporin resistant *E. coli* isolates from the fecal microbiota of captive wild mammals (llama, lion, hyena, and tiger) adds to the growing evidence that wildlife, including zoo and farmed exotic animals, can act as reservoirs of clinically relevant AMR determinants.

Similar studies involving zoo mammals and free-ranging wildlife have identified multidrug-resistant (MDR) *E. coli* and other Enterobacterales in carnivores and ungulates [9,10]. These findings indicate that such resistance is often present at low-to-moderate prevalence, yet the resistance profiles observed are concerning [10].

The preserved susceptibility of these isolates to carbapenems, colistin, and tigecycline suggests that, in this setting, resistance to key last-resort agents has not yet emerged or become established; nevertheless, the coexistence of third-generation cephalosporin resistance with multidrug resistance in wildlife-associated *E. coli* mirrors patterns seen in human and livestock compartments and fits into a One Health scenario where resistant clones and plasmids may circulate across species and environments [11]. Overall, the phenotypic resistance profiles were concordant with the detected resistome and chromosomal determinants, supporting a consistent phenotype–genotype relationship across the antimicrobial classes tested.

From a genomic standpoint, the assemblies obtained are highly consistent with the known genomic architecture of *E. coli*, supporting the reliability of downstream in silico analyses.

The gene content falls squarely within the expected range for this species, indicating that the four genomes are broadly complete in functional terms. Beyond these basic metrics, the high numbers of annotated AMR genes (61–68 CARD hits) and virulence-associated loci (114–131 VFDB hits) underscore that these wildlife isolates are not innocuous commensals but are genetically equipped to withstand multiple antimicrobial classes and to express a broad virulence repertoire, as has been increasingly described for MDR *E. coli* from wild animals in other regions [12].

The detection of 142–146 metal-resistance genes (BacMet) and nearly 1000 predicted transporters (TCDB) per genome is also noteworthy. Metal-resistance determinants and efflux systems are increasingly recognized as important components of the resistome: they can enhance bacterial fitness in contaminated environments and contribute to co-selection of antibiotic resistance when metals, biocides, and antibiotics co-occur [13,14].

The four wildlife isolates clustered into two well-known extraintestinal *E. coli* lineages, highlighting the integration of these animals into broader One Health transmission networks. The llama (Ec1) and tiger (Ec4) strains belonged to ST162, phylogroup B1, serotype O134:H19, whereas the lion (Ec2) and hyena (Ec3) carried ST69, phylogroup D, serotype O15:H18.

ST162 is a successful, globally disseminated lineage that has been reported in humans, livestock, companion animals, and wildlife, often in association with multidrug resistance and ESBL or AmpC production, including in fecal *E. coli* from wild mammals [11,15,16].

Similarly, ST69 is a pandemic ExPEC clone classically associated with urinary tract and bloodstream infections in humans and has also been detected in companion animals and environmental sources, underscoring its broad ecological range [17,18]. The detection of these two high-risk clonal lineages in carnivores and a camelid kept in a private wildlife farm suggests that wild or captive wild animals can act as sentinels or secondary reservoirs for globally circulating ExPEC lineages, likely reflecting spillovers from human, domestic animal, or environmental sources and fitting squarely within a One Health framework.

Higher-resolution genotyping confirmed the presence of two distinct genomic backgrounds. Ribosomal MLST assigned rST 1544 to the ST162/B1 strains and rST 2135 to the ST69/D strains, consistent with the ability of rMLST to resolve fine-scale clonal structure [19]. Using the 2513-locus *Escherichia/Shigella* cgMLST scheme, reliable cgSTs were obtained for Ec1 (cgST 207265) and Ec3 (cgST 134012), confirming that they belong to distinct core-genome lineages. Although Ec2 and Ec4 could not be assigned cgSTs, species identity as *E. coli* and the main β-lactamase genes were independently confirmed from raw reads by KmerResistance/KmerFinder, supporting the robustness of the in silico typing pipeline.

CRISPR–Cas profiles indicated lineage-specific differences in adaptive immunity to mobile elements: Ec3 (ST69, hyena) and Ec4 (ST162, tiger) carried a complete type I-E CRISPR–Cas locus with two large CRISPR arrays, whereas Ec1 and Ec2 harbored only short orphan CRISPR candidates without *cas* genes. This heterogeneity mirrors previous observations that CRISPR–Cas systems in *E. coli* are variably maintained across phylogroups and ExPEC lineages and do not necessarily prevent acquisition of mobile resistance determinants [20]. In our isolates, both CRISPR-positive and CRISPR-poor genomes carried numerous resistance and virulence genes, suggesting that CRISPR–Cas variation more likely reflects differences in past phage and plasmid exposure than a simple barrier to horizontal gene transfer. Together, these phylogenetic and in silico typing data show that captive wild animals can harbor globally disseminated ExPEC-like clones with diverse genome architectures, underlining their relevance for One Health surveillance of antimicrobial resistance.

The fimbrial and fumC–fimH backgrounds further support the ExPEC potential of these isolates. FimTyper identified fimH32 in the ST162/B1 genomes (Ec1/Ec4) and fimH27 in the ST69/D genomes (Ec2/Ec3), in combination with fumC65 and fumC35, respectively. These fumC–fimH combinations have been reported in ExPEC-associated lineages, including ST162 and ST69 clones causing extraintestinal infections in humans and animals, and are frequently linked to multidrug-resistant or ESBL-producing strains [21,22]. The presence of such ExPEC-associated fimH alleles in wildlife isolates, together with their virulence gene profiles, suggests that these strains could, at least in principle, cause disease if transmitted to humans or domestic animals, reinforcing concerns about zoonotic and environmental circulation of ExPEC lineages.

The resistome of the four wildlife *E. coli* isolates was highly complex, combining multiple acquired AMR genes with target-site and regulatory mutations. The ubiquitous presence of *bla*_TEM-1B_, together with sulfonamide and trimethoprim determinants (*sul2*, *dfrA14/dfrA17*) and several aminoglycoside-modifying enzymes (*aadA5*, *aph(6)-Id*, *aph (3″)-Ib*), mirrors the classical MDR backbone reported in ESBL/AmpC-producing *E. coli* from humans, livestock, and companion animals [23]. Detection of *tet*(B) in the ST162/B1 isolates (llama and tiger) is consistent with the strong ecological footprint of tetracycline use in food-producing animals and the environment, where *tet* genes are among the most widespread resistance determinants and frequently spill over into wildlife microbiota [24]. In this context, the identification of a similar MDR gene complement in wildlife-associated carnivores in Tunisia suggests that these animals are integrating AMR signals from anthropogenic sources (feed, water, human contact), reinforcing the One Health nature of this reservoir.

The β-lactam resistance profile is particularly concerning from a clinical and public-health perspective. The ST69/phylogroup D isolates (lion and hyena) carried *bla*_CTX-M-15_, one of the most prevalent ESBLs globally in *E. coli* from humans, livestock, and the environment, including Tunisia [25,26,27]. In contrast, the ST162/B1 strains (llama and tiger) harbored *bla*_CMY-2_, a plasmid-mediated AmpC enzyme widely reported in poultry, companion animals, and wildlife, and increasingly implicated in human infections [28]. Co-occurrence of these broad-spectrum cephalosporinases with *bla*_TEM-1B_ and narrow-spectrum β-lactamases (*bla*_EC-8_/*bla*_EC-18_) illustrates the layered nature of β-lactam resistance in these isolates. Similar combinations of CTX-M-type or CMY-2 with TEM-1 have been described in ExPEC recovered from humans, poultry, and wild birds and are often plasmid-borne, facilitating horizontal transfer across host species [29].

The fluoroquinolone resistance determinants also show a “human-like” pattern that is worrisome in a wildlife setting. In the ST162 isolates, the double mutations *gyrA* S83L/D87N combined with *parC* S80I correspond to the canonical QRDR profile associated with high-level fluoroquinolone resistance in clinical *E. coli* [30]. By contrast, the ST69 isolates retained wild-type *gyrA/parC* but carried *qnrS1*, a plasmid-mediated quinolone resistance (PMQR) gene that confers low-level resistance and strongly promotes selection of QRDR mutants under fluoroquinolone exposure [31]. This pattern-PMQR alone in some lineages and full QRDR mutations in others-is in line with previous One Health studies showing stepwise evolution of fluoroquinolone resistance across humans, domestic animals, and wildlife, and again suggests that our wildlife-associated isolates participate in the same global resistance network.

Additional chromosomal mutations (GlpT E448K and *cyaA* S352T) may impair fosfomycin uptake, while PBP3 changes resemble cephalosporin-resistant variants. Variants in AcrR, MarR, and oxidative stress regulators (SoxR/S) likely promote efflux-mediated multidrug resistance. Together, these findings highlight captive wildlife as reservoirs and potential amplifiers of clinically relevant resistance traits.

The genomic profiles of the four *E. coli* isolates reveal a comprehensive set of virulence-associated traits, plasmids, and mobile genetic elements characteristic of extraintestinal pathogenic *E. coli* (ExPEC). All strains carried adhesins, including the type 1 fimbrial adhesin, multiple other fimbrial subunits, curli fiber genes *(csgA*), and the outer membrane protease ompT, which are associated with adhesion/biofilm formation to host tissues and biofilm formation [32]. Additionally, these isolates harbored a range of iron-acquisition systems, commonly associated with ExPEC, reflecting their adaptation to iron-limited environments encountered in both animal hosts and humans. Several toxin-encoding and serum-resistance–associated genes were also detected, which are commonly linked to persistence and survival in extraintestinal environments. Overall, each isolate possessed a suite of ExPEC-linked virulence factors, including adhesins, siderophores, toxins, and immune evasion genes, commonly found in pathogenic human strains [32]. Importantly, these findings align with previous studies of wildlife-derived *E. coli*, which often show a mixture of commensal and ExPEC traits, underscoring the complex ecology of these bacteria [33].

Conjugative plasmids are well recognized as major vehicles for the horizontal dissemination of ESBL and plasmid-mediated AmpC determinants in *E. coli*, often carrying additional resistance modules that enable co-selection under multiple antimicrobial exposures [34]. In this context, the successful transfer of *bla*_CMY-2_ and *bla*_CTX-M-15_ to an *E. coli* J53 recipient in our mating-out assays supports that the key β-lactam resistance determinants identified in these wildlife isolates are mobilizable rather than strictly clonal. Co-transfer of tetracycline/chloramphenicol and, in some transconjugants, trimethoprim–sulfamethoxazole further aligns with the frequent clustering of multiple resistance determinants on the same conjugative elements, which can sustain MDR even when selective pressure is exerted by non-β-lactam agents [34]. Finally, the appearance of ciprofloxacin resistance alongside ESBL transfer in ST69-derived transconjugants is consistent with reports that plasmid-mediated quinolone resistance determinants (*qnr* genes) commonly co-occur with ESBLs, facilitating persistence and spread under fluoroquinolone exposure and promoting the stepwise selection of higher-level quinolone resistance [35]. Collectively, these findings reinforce the epidemiological relevance of the resistome detected here by providing functional evidence for horizontal gene transfer potential, while underscoring that defining the precise plasmid backbones and genetic contexts will require long-read sequencing approaches.

PathogenFinder predicted a high probability of human pathogenicity for all genomes (≥0.93), with hundreds of proteins matching known pathogenic *E. coli* families and very few matching non-pathogenic ones [36]. However, these outputs are in silico estimates based on gene/protein-family content and do not demonstrate gene expression or virulence phenotypes in vitro or in vivo. Therefore, the pathogenic potential inferred here should be interpreted as genomic evidence consistent with an ExPEC-like virulence repertoire and as a rationale for future functional validation (e.g., cell-based adhesion/invasion assays and complementary pathogenicity readouts), as suggested in related wildlife studies [12].

The ST162/B1 isolates carried a complex plasmidome comprising IncFIB, IncFIC(FII), IncI1, and small Col-type plasmids, while the ST69/D isolates exhibited a simpler profile with IncY and Col(MG828) plasmids. This variety of incompatibility groups and presence of common plasmid types are typical for *E. coli* from diverse sources [37]. Insertion sequences (IS) were abundant across all genomes, reflecting a high potential for horizontal gene mobilization. Notably, IS26 is known to drive genetic rearrangements and mobilize resistance and virulence genes across plasmids and chromosomes [37]. Together, the plasmidome and IS content indicate that these isolates are well-equipped to acquire and disseminate genes within microbial communities spanning human, domestic animal, and wildlife environments.

Comparing our results to other wildlife-derived *E. coli* studies reveal both similarities and notable differences by host species. As in our work, many free-ranging bird and mammal studies report low levels of clinical antimicrobial resistance but detect typical ExPEC virulence sets. For instance, wild bird surveys have repeatedly found pandemic human clones (ST131, ST69) and ESBL enzymes carrying ExPEC virulence genes, despite minimal direct exposure to antibiotics. Likewise, wild boars and other mammals often carry phylogroup B1 strains with IncF plasmids and siderophores, similar to our ST162 isolates. However, host ecology can bias the lineage distribution: scavenging birds frequently harbor *E. coli* lineages that overlap with human or livestock sources, whereas more isolated or herbivorous wildlife may carry strains more typical of the local environment. The fact that one of our isolates (ST162/B1) came from a llama and displayed a human-linked virulence profile is a clear example of this effect.

These host-specific patterns underscore the importance of wildlife sampling in understanding pathogen transmission. When various species harbor distinct *E. coli* lineages, it indicates different exposure routes or transmission networks.

The cgMLST-based minimum-spanning trees showed that the Tunisian isolates are embedded within internationally disseminated ST69 and ST162 lineages rather than forming separate, wildlife-restricted branches. Ec2 and Ec3 (ST69) clustered tightly together and were most closely related to food and animal isolates from Ghana and the UK, while Ec1 and Ec4 (ST162) occupied a central position in a star-like network of predominantly animal and food isolates from Europe, Asia, and South America, separated from their nearest neighbors by only a few dozen to ~100 alleles. These patterns indicate that the wildlife isolates share recent common ancestry with strains circulating in human, livestock, and food compartments worldwide, supporting the view that ST69 and ST162 are part of a global gene pool that can move across species and geographic boundaries. The predominance of animal and food sources among closely related ST162 genomes, and the proximity of our ST69 isolates to clinical strains, further suggest that both food-production systems and human clinical settings may act as key reservoirs from which ExPEC lineages can spill over into captive wildlife.

Beyond our captive-wildlife isolates, ST162 and ST69 lineages have been reported across multiple One Health compartments in Tunisia. Notably, ESBL-producing *E. coli* recovered from healthy livestock and municipal wastewater included both ST69 and ST162, supporting circulation at the human–animal–environment interface [38]. Wildlife may also contribute: ESC-resistant *E. coli* from migratory European starlings in Tunisia encompassed diverse clones, including ST162, and the authors explicitly frame this as a One Health concern [39]. In poultry-linked settings, ST69 has likewise been documented among Tunisian ESBL *E. coli* collections [40], and clinical series describe ST69 as a globally disseminated ExPEC lineage in Tunisia. Together, these reports reinforce that the resistant clones observed here likely reflect broader cross-sectoral circulation, underscoring the value of integrated One Health surveillance [41].

From a One Health perspective, these genomic findings suggest that captive wildlife may act as sentinels of clinically relevant MDR/ExPEC-like *E. coli* circulating in human-impacted ecosystems. Nevertheless, our inferences are based on gene content and comparative genomics, and neither transmission directionality nor virulence phenotypes can be demonstrated without dedicated environmental/human-interface sampling and functional assays. Integrated One Health surveillance remains warranted to better resolve dissemination routes and public health relevance.

Limitations. This study has limitations that should be acknowledged. Only 30 fecal samples from 14 captive wild animals were screened, yielding four 3GC-resistant *E. coli* isolates; therefore, statistical inference is limited and the findings cannot be considered ecologically representative of captive wildlife in Tunisia. In addition, virulence and pathogenicity were inferred from in silico predictions (virulence gene detection and PathogenFinder scoring). We did not perform expression studies or cell-based/in vivo pathogenicity assays; therefore, the actual virulence phenotype remains to be experimentally confirmed. Consequently, our results should be interpreted as an exploratory, WGS-based characterization of clinically relevant ESBL/AmpC lineages rather than an estimate of prevalence. Larger, multi-site and longitudinal studies are needed to better define the diversity, frequency and transmission pathways of MDR *E. coli* at the human–animal–environment interface.

Importantly, our study did not include environmental sampling (e.g., soil, water, feed, or fomites) or data from animal caretakers; therefore, potential transmission pathways at the human–animal–environment interface cannot be resolved. Because sampling was cross-sectional, temporal trends in AMR could not be assessed; longitudinal, multi-year surveillance across additional sites and host species is warranted to monitor changes over time. Moreover, sampling was confined to a single private wildlife farm and did not include free-ranging wildlife or other habitats, limiting ecological representativeness. Future One Health studies should adopt multi-site and longitudinal designs that simultaneously sample wildlife, environmental matrices and relevant human interfaces to better characterize sources, directionality and routes of dissemination of MDR *E. coli*.

Sampling was performed at a single time point in 2018; therefore, temporal trends and potential seasonal variation in MDR *E. coli* carriage could not be assessed. Longitudinal, multi-season and multi-year surveillance will be required to determine whether the detected ESBL/AmpC lineages persist over time, fluctuate seasonally, or reflect transient introductions.

Future studies should expand sampling to include free-ranging wildlife, multiple facilities/sites, and a wider diversity of host species to improve ecological representativeness and enable more robust comparisons across settings. In particular, larger multi-site and longitudinal designs are needed to better delineate the diversity and circulation of MDR/ESBL/AmpC-producing *E. coli* at the human–animal–environment interface and to clarify potential transmission pathways between captive and free-ranging populations.

In addition, because our genomic analyses relied on Illumina short-read assemblies, we could not reconstruct complete/closed plasmids or fully resolve the genetic contexts of resistance determinants. Future work using long-read sequencing (Oxford Nanopore/PacBio), ideally in hybrid assemblies with Illumina data, will be required to close plasmids and precisely map mobile genetic elements and resistance/virulence loci

Despite these limitations, our findings provide a genomic snapshot of clinically relevant MDR *E. coli* lineages in captive wildlife and underscore priorities for future surveillance.

## 4. Materials and Methods

### 4.1. Bacterial Isolation and Antimicrobial Susceptibility Testing

Fourteen wild animals housed in a private wildlife farm in northeastern Tunisia were screened in 2018. A total of 30 fresh fecal droppings were sampled on the ground using sterile cotton swabs, including samples from tiger (*n* = 4), lion (*n* = 5), hyena (*n* = 4), Watusi cattle (*n* = 4), dwarf goat (*n* = 7), and llama (*n* = 6).

Samples were processed within 24 h of collection after the swabs were promptly put into a Brain Heart Infusion (BHI; Oxoid, Basingstoke, UK) and brought to the lab in insulated cartons with cold packs.

After incubating BHI tubes at 37 °C for 18–24 h, aliquots of the enriched cultures were streaked onto deoxycholate lactose agar (GDL; Oxoid, Basingstoke, UK) supplemented with cefotaxime (2 µg/mL; Sigma-Aldrich, St. Louis, MO, USA) in order to select third-generation cephalosporin-resistant Gram-negative bacteria.

After incubation at 37 °C for 18–24 h, colonies with morphology compatible with *E. coli* were subcultured and identified by conventional biochemical tests, including the API 20E system (bioMérieux, Marcy-l’Étoile, France).

Antimicrobial susceptibility testing was performed following EUCAST recommendations. Disk diffusion on Mueller–Hinton agar (Bio-Rad, Marnes-la-Coquette, France) was used for β-lactams (ampicillin, amoxicillin–clavulanic acid, ticarcillin, ticarcillin–clavulanic acid, ertapenem, imipenem, meropenem, cephalothin, cefoxitin, cefotaxime, ceftazidime, cefepime, aztreonam), aminoglycosides (tobramycin, netilmicin, amikacin), quinolones/fluoroquinolones (nalidixic acid, norfloxacin, ciprofloxacin), tetracyclines (tetracycline, minocycline), and other agents (fosfomycin, chloramphenicol, trimethoprim–sulfamethoxazole).

Colistin susceptibility was determined by broth microdilution MIC (reference method), and tigecycline MIC was determined by gradient diffusion (Etest; bioMérieux, Marcy-l’Étoile, France). In addition, MICs were determined by broth microdilution for ampicillin, cefotaxime, amikacin, ciprofloxacin, imipenem, and ertapenem. Disk diffusion zone diameters and MIC values were interpreted according to EUCAST guidelines.

Isolates were screened for ESBL-phenotype by double-disk synergy test (DDST) with cefotaxime, ceftazidime, and amoxicillin-clavulanic acid disks. Isolates showing a negative-ESBLphenotype with resistance to amoxicillin-clavulanic acid and to cefoxitin were classified as AmpC producers. To aid detection in the presence of AmpC activity, the DDST was repeated on Mueller–Hinton agar supplemented with cloxacillin (75 mg/L; Sigma-Aldrich, St. Louis, MO, USA) as an AmpC inhibitor.

### 4.2. DNA Extraction, Library Preparation and Whole-Genome Sequencing

Genomic DNA was extracted from overnight cultures of the *E. coli* isolates using the DNeasy Blood and Tissue Kit (Qiagen, Hilden, Germany), following the manufacturer’s instructions.

A260/280 and A260/230 ratios were measured using a NanoDrop spectrophotometer (Thermo Fisher Scientific, Waltham, MA, USA) to determine the extracted DNA’s purity.

The Qubit dsDNA assay kit and a Qubit fluorometer (Thermo Fisher Scientific; Waltham, MA, USA) were then used to quantify DNA concentrations fluorometrically, and the results were modified to meet input requirements for library construction.

Whole-genome sequencing (WGS) libraries were prepared using the Illumina DNA Prep kit (Illumina, San Diego, CA, USA) according to the manufacturer’s instructions. Indexed libraries were pooled equimolarly and sequenced on an Illumina iSeq 100 (Illumina, San Diego, CA, USA).

### 4.3. Read Quality Control and De Novo Assembly

Raw reads were subjected to quality assessment using FastQC (Galaxy platform; Version 0.74+galaxy1; https://usegalaxy.eu (accessed on 15 November 2025)) and overall read quality and per-base sequence metrics were inspected to confirm the absence of major artifacts.

The Proksee web server (https://proksee.ca (accessed on 17 November 2025)), which offers a short-read assembler designed for bacterial genomes with default parameters, was then used to de novo assemble the Illumina reads.

For each isolate, the resulting draft genome was further evaluated based on assembly statistics (genome size, GC content, number of contigs, N50, and L50). Raw sequence reads for all four isolates have been deposited in the NCBI Sequence Read Archive (SRA).

Draft genomes were assembled using the Comprehensive Genome Analysis pipeline of BV-BRC (v3.55.17; https://www.bv-brc.org/app/ComprehensiveGenomeAnalysis (accessed on 17 November 2025)), which selects an appropriate assembler and optimizes assembly parameters based on read characteristics. The BV-BRC assemblies and associated metrics were used as an independent quality control of the Proksee-based assemblies.

### 4.4. Genome Annotation and CRISPR Analysis

Functional annotation of the Proksee assemblies (https://proksee.ca (accessed on 17 November 2025)) was primarily performed using Bakta (tool v1.1.0; Bakta v1.8.2, DB v5.0—Light; https://proksee.ca/tools/bakta (accessed on 17 November 2025)), which predicts coding sequences, RNA genes, and functional attributes for bacterial genomes. Prokka (tool v1.2.0; Prokka v1.14.6; https://proksee.ca/tools/prokka (accessed on 18 November 2025)) was additionally run on the same assemblies to cross-check gene predictions and functional assignments, and discrepancies were manually inspected. As a complementary approach, genomes were annotated with the RASTtk pipeline within BV-BRC (https://www.bv-brc.org/ (accessed on 18 November 2025)), providing independent counts of coding sequences, tRNAs, and rRNAs and assigning genes to subsystems and PATRIC protein families. Global genome features (AMR, virulence, metal-resistance genes, and transporters) were extracted from the BV-BRC reports.

CRISPRCasFinder (as implemented in the Proksee platform; v1.1.0: https://proksee.ca/tools/crispr_cas_finder (accessed on 24 November 2025)) was used with default parameters to identify putative CRISPR arrays and related *cas* genes.

### 4.5. In Silico Typing and Phylogenetic Context

Classical seven-locus MLST, O:H serotypes, and phylogroups were obtained by uploading assemblies to EnteroBase (*Escherichia/Shigella* database) (https://enterobase.warwick.ac.uk/species/index/ecoli (accessed on 18 November 2025)). Phylogroups were inferred using the in silico Clermont typing scheme implemented in EnteroBase.

To offer a reliable, high-resolution indicator of the genomic backdrop, ribosomal sequence types (rSTs) were obtained from the EnteroBase rMLST method.

To further describe the ExPEC-related lineages, CH clonotypes (fumC–fimH pairings) were assigned using CHTyper 1.0 (https://cge.food.dtu.dk/services/CHTyper/ (accessed on 21 November 2025)) with the same identity cutoff, and fimH alleles were identified using FimTyper 1.0 (Center for Genomic Epidemiology; https://cge.food.dtu.dk/services/FimTyper/ (accessed on 18 November 2025)) with a minimum identity threshold of 95%.

Core-genome multilocus sequence typing (cgMLST) was used to further characterize genomic relatedness. The *Escherichia*/*Shigella* 2513-locus scheme with default parameters was used to analyze draft genome assemblies uploaded to the cgMLSTFinder 1.2 tool (software version 1.0.1; CGE) (https://cge.food.dtu.dk/services/cgMLSTFinder/ (accessed on 21 November 2025)). Alleles were assigned at each locus, and a core-genome sequence type (cgST) was called based on the allele profile, treating loci without an assigned allele (including hypothetical novel alleles) as missing data. Allele calling depends on locus completeness in draft assemblies and on identity/coverage thresholds used by the calling algorithm. When cgMLSTFinder did not return a callable profile for some assemblies, we used the PubMLST implementation of the *Escherichia/Shigella* cgMLST scheme (https://pubmlst.org/organisms/escherichia-spp (accessed on 19 November 2025)) to obtain cgMLST profiles and build minimum-spanning trees for comparative analysis. Species identity as *E. coli* and the presence of acquired resistance genes were additionally confirmed from raw reads using KmerResistance (CGE; https://cge.food.dtu.dk/services/KmerResistance/ (accessed on 21 November 2025)), with species determination based on maximum query coverage, a minimum identity threshold of 70%, and a depth-correction threshold of 10%. KmerFinder 3.2 (software version 3.0.2; https://cge.food.dtu.dk/services/KmerFinder-3.2 (accessed on 21 November 2025)) was also used for k-mer-based species confirmation with default parameters.

### 4.6. Resistome, Virulome and Mobile Genetic Elements

ResFinder (CGE; https://cge.food.dtu.dk/services/ResFinder/ (accessed on 19 November 2025)) was used to identify acquired antimicrobial resistance genes from assemblies. For both acquired resistance genes and chromosomal point mutations, a minimum identity threshold of 90% and a minimum alignment length of 60% of the reference gene were required.

The Proksee pipeline (https://proksee.ca (accessed on 19 November 2025)), which offered a supplemental annotation of resistance determinants, such as efflux pumps and regulatory genes, was used to combine the results with those from the Comprehensive Antibiotic Resistance Database (CARD; https://card.mcmaster.ca/ (accessed on 19 November 2025)).

Virulence-associated genes were predicted with VirulenceFinder 2.0 (software version 2.0.5; CGE; https://cge.food.dtu.dk/services/VirulenceFinder/ (accessed on 21 November 2025)), using a minimum identity threshold of 90% and a minimum gene length of 60% of the reference. Virulence factor annotations from VFDB (http://www.mgc.ac.cn/VFs/ (accessed on 21 November 2025)), as reported by BV-BRC (https://www.bv-brc.org/ (accessed on 17 November 2025)) specialty-gene analysis, were used as an additional source of virulence-related information.

Plasmid replicon types were determined using PlasmidFinder 2.1 (software version 2.0.1; CGE; https://cge.food.dtu.dk/services/PlasmidFinder/ (accessed on 20 November 2025)), restricted to the Enterobacteriales database, with thresholds of 95% minimum identity and 60% minimum coverage. For plasmid sequence types, the pMLST 2.0 tool (software version 0.1.0; CGE; https://cge.food.dtu.dk/services/pMLST/ (accessed on 20 November 2025)) was applied to relevant incompatibility groups using default settings.

The MGE process (software version v1.0.3, database version v1.0.2; CGE; https://cge.food.dtu.dk/services/MobileElementFinder/ (accessed on 21 November 2025)) was used to characterize mobile genetic elements, and ISfinder (https://isfinder.biotoul.fr/ (accessed on 21 November 2025)) was used to manually curate and rename insertion sequences in accordance with conventional nomenclature.

### 4.7. Transfer of Resistance Determinants

Genetic support was studied by mating-out assays for all strains. Each wildlife isolate was used as a donor and the sodium azide-resistant *E. coli* J53 strain was used as the recipient. Putative transconjugants were selected on Mueller–Hinton agar (Bio-Rad, Marnes-la-Coquette, France) containing sodium azide (100 mg/L; Sigma-Aldrich, St. Louis, MO, USA) and cefotaxime (2 mg/L; Sigma-Aldrich, St. Louis, MO, USA). Transconjugants resistance profiles were tested by disk diffusion method and PCR.

### 4.8. In Silico Prediction of Human Pathogenic Potential

The probability that each isolate could act as a human pathogen was estimated using the PathogenFinder web tool (CGE; https://cge.food.dtu.dk/services/PathogenFinder/ (accessed on 20 November 2025)), with automatic model selection based on the taxonomic affiliation of the query genome and default thresholds (minimum identity 100%; Z-score threshold 25.37).

PathogenFinder, which compares genome-encoded protein families to reference sets from human-pathogenic and non-pathogenic bacteria and returns a probability score between 0 and 1 of being a human pathogen, the number of matched protein families and their classification as pathogenic or non-pathogenic, and the percentage of the genome covered by protein families associated with pathogenic bacteria, received the translated coding sequences for each genome assembly.

These outputs were interpreted as in silico estimates and were not experimentally validated by expression or cell-based assays.

### 4.9. Identification of Closely Related Genomes

Genomes were retrieved from the *Escherichia* spp. PubMLST database (https://pubmlst.org/organisms/escherichia-spp (accessed on 23 November 2025)).

Initially, the 15,512 full genomes (>4 Mb) available in the database were analyzed.

From these, genomes assigned to ST69 (*n* = 309) and ST162 (*n* = 102) were selected. To identify the closest genetically related isolates and to improve phylogenetic visualization, only genomes representing the most informative genomic distances were retained. This filtering step resulted in a final dataset of 21 ST69 and 33 ST162 genomes included in the comparative analysis (Supplementary Table S1). Core genome comparisons (2513 loci; *E. coli* cgMLST v1.0) were performed using the GrapeTree tool implemented in PubMLST (https://pubmlst.org/analysis/grapetree (accessed on 23 November 2025)).

### 4.10. Use of Generative AI

During the preparation of this manuscript, the authors used ChatGPT (OpenAI, San Francisco, CA, USA; https://chat.openai.com (accessed on 27 December 2025)) solely for English-language editing (grammar, wording, and clarity).

## 5. Conclusions

In this study, we performed phenotypic and whole-genome characterization of multidrug-resistant, third-generation cephalosporin-resistant *E. coli* isolates from captive wild mammals in Tunisia. Our findings revealed two prominent ExPEC lineages, ST162 and ST69, harboring CMY-2 and CTX-M-15 enzymes, respectively, alongside a broad arsenal of virulence and resistance genes, mobile genetic elements, and plasmids. The isolates demonstrated high predicted pathogenicity and shared genomic signatures with clinically relevant human strains. Collectively, these results suggest that captive wildlife may act as potential reservoirs and sentinels of clinically relevant MDR/ExPEC-like *E. coli*, as inferred from genomic analyses. Given the interconnectedness of ecosystems, humans, and animals, our findings reinforce the need for integrated One Health surveillance and containment strategies.

## Figures and Tables

**Table 1:** Antimicrobial susceptibility profiles of the four MDR *E. coli* isolates recovered from captive wildlife.

Antimicrobial Class	Antibiotic	Ec1	Ec4	Ec2	Ec3
Penicillins	Ampicillin	R	R	R	R
	Ticarcillin	R	R	R	R
Betalactam/beta-lactamase inhibitor combinations	Amoxicillin-clavulanic acid	R	R	I	I
	Ticarcillin-clavulanic acid	R	R	I	I
Cephalosporins/monobactam	Cephalothin (1st-gen)	R	R	R	R
	Cefoxitin (cephamycin)	R	R	S	S
	Cefotaxime (3rd-gen)	R	R	R	R
	Ceftazidime (3rd-gen)	R	R	R	R
	Cefepime (4th-gen)	R	R	R	R
	Aztreonam (monobactam)	R	R	R	R
Carbapenems	Ertapenem	S	S	S	S
	Imipenem	S	S	S	S
	Meropenem	S	S	S	S
Aminoglycosides	Tobramycin	R	R	R	R
	Netilmicin	R	R	I	R
	Amikacin	S	S	S	S
Quinolones/fluoroquinolones	Nalidixic acid	R	R	R	R
	Norfloxacin	R	R	R	R
	Ciprofloxacin	R	R	R	R
Tetracyclines/glycylcycline	Tetracycline	R	R	R	R
	Minocycline	R	R	I	I
	Tigecycline	S	S	S	S
Others	Fosfomycin	I	I	I	I
	Chloramphenicol	R	R	R	R
	Trimethoprim-sulfamethoxazole	R	R	R	R

S, susceptible; I, susceptible, increased exposure (EUCAST); R, resistant; 1st-gen, first generation; 3rd-gen, third generation; 4th-gen, fourth generation; MDR, multidrug-resistant.

**Table 2:** General genomic features of third-generation cephalosporin-resistant *Escherichia coli* strains isolated from captive wild mammals in Tunisia.

Isolate ID	Host	Genome Length (bp)	GC (%)	Contigs	N50 (bp)	L50	CDS	tRNA	rRNA	AMR Genes (CARD)	Virulence Genes (VFDB)	Metal-Resistance Genes (BacMet)	Transporters (TCDB)
Ec1	Llama	5,017,117	50.55	81	188,611	9	4980	75	4	68	120	145	973
Ec2	Lion	4,951,530	50.64	73	251,976	8	4794	80	3	62	114	144	945
Ec3	Hyena	4,918,706	50.62	68	180,087	9	4735	76	3	61	114	142	942
Ec4	Tiger	5,025,665	50.55	97	193,171	8	5016	75	5	68	131	146	977

ST, sequence type; GC, guanine–cytosine; bp, base pairs; N50, length of the shortest contig at 50% of the total genome length; L50, smallest number of contigs whose cumulative length accounts for 50% of the genome length; CDS, coding DNA sequences; tRNA, transfer RNA; rRNA, ribosomal RNA; AMR, antimicrobial resistance; CARD, Comprehensive Antibiotic Resistance Database; VFDB, Virulence Factors of Pathogenic Bacteria database; BacMet, Bacterial Metal Resistance Genes database; TCDB, Transporter Classification Database.

**Table 3:** Antimicrobial resistance genes, key chromosomal mutations, virulence factors, plasmid replicons and insertion sequences in third-generation cephalosporin-resistant *Escherichia coli* strains isolated from captive wild mammals in Tunisia.

Sample ID	SRA Run Accession	Biosample Accession	Sequence Type	Serotype	Phylogroup	Antibiotic Resistance Genes	Chromosomal Mutations	Virulence Genes	Plasmids	Insertion Sequences
Ec1	SRR36138962	SAMN53358001	ST162	O134:H19	B1	*vanG*, *bla*_CMY-2_, *bla*_TEM-1B_, *aadA5*, *dfrA17*, *catA1*, *tet*(B), *bla*_EC-18_, *aph*(6)-Id, *sul2*, *aph*(3′)-Ia, *aph*(3″)-Ib	gyrA (S83L, D87N), parC (S80I), GlpT (E448K), PBP3 (D350N, S357N), AcrAB-TolC with AcrR mutation, AcrAB-TolC with MarR mutations (Y137H, G103S), soxR and soxS mutations	*anr*, *astA*, *cib*, *csgA*, *cvaC*, *etsC*, *fdeC*, *fimH32*, *fumC65*, *gad*, *hlyE*, *hlyF*, *hra*, *iroN*, *iss*, *iucC*, *iutA*, *lpfA*, *mchF*, *nlpI*, *ompT*, *papC*, *sitA*, *terC*, *traJ*, *traT*, *yehA*, *yehB*, *yehC*, *yehD*, *yghJ*	ColpVC, IncFIB, IncFIC(FII), IncI1-I(Alpha), IncQ1	ISEc9, IS629, MITEEc1 (IS630), IS26
Ec2	SRR36138961	SAMN53358002	ST69	O15:H18	D	*vanG*, *bla*_EC-8_, *qnrS1*, *bla*_CTX-M-15_, *bla*_TEM-1B_, *aph*(6)-Id, *aph* (3″)-Ib, *sul2*, *dfrA14*	GlpT (E448K),cyaA (S352T), PBP3 (D350N, S357N), AcrAB-TolC with AcrR mutation, AcrAB-TolC with MarR mutations (Y137H, G103S), soxS, soxR	*csgA*, *fdeC*, *fimH27*, *fumC35*, *gad*, *hlyE*, *iss*, *lpfA*, *nlpI*, *ompT*, *sitA*, *terC*, *yehA*, *yehB*, *yehC*, *yehD*, *yghJ*, *AslA*, *chuA*, *eilA*, *fyuA*, *hha*, *irp2*, *kpsE*, *kpsMIII_K96*	IncY	ISEc9, ISKpn19, MITEEc1, ISEc46, ISEc38, IS4, ISSfl10, IS629, ISEc31, IS26
Ec3	SRR36138960	SAMN53358003	ST69	O15:H18	D	*vanG*, *bla*_EC-8_, *sul2*, *aph* (3″)-Ib, *aph* (6)-Id, *bla*_TEM-1B_, *bla*_CTX-M-15_, *qnrS1*, *dfrA14*	GlpT (E448K), cyaA (S352T), PBP3 (D350N, S357N), AcrAB-TolC with AcrR, soxS, soxR, AcrAB-TolC with MarR mutations (Y137H, G103S)	*csgA*, *fdeC*, *fimH27*, *fumC35*, *gad*, *hlyE*, *iss*, *lpfA*, *nlpI*, *ompT*, *sitA*, *terC*, *yehA*, *yehB*, *yehC*, *yehD*, *yghJ*, *AslA*, *chuA*, *eilA*, *fyuA*, *hha*, *irp2*, *kpsE*, *kpsMIII_K96*	Col(MG828), IncY	ISEc9, MITEEc1, ISEc46, IS4, ISEc38, ISSfl10, IS629, ISEc31, IS26
Ec4	SRR36138959	SAMN53358004	ST162	O134:H19	B1	*vanG*, *bla*_CMY-2_, *bla*_TEM-1B_, *bla*_EC-18_, *catA1*, *aadA5*, *tet*(B), *sul2*, *aph* (6)-Id, *aph* (3′)-Ia, *dfrA17*	GlpT (E448K), gyrA (D87N, S83L), PBP3 (D350N, S357N), parC (S80I), AcrAB-TolC with AcrR mutation, soxR, soxS, AcrAB-TolC with MarR mutations (Y137H, G103S)	*anr*, *astA*, *cib*, *csgA*, *cvaC*, *etsC*, *fdeC*, *fimH32*, *fumC65*, *gad*, *hlyE*, *hlyF*, *hra*, *iroN*, *iss*, *iucC*, *iutA*, *lpfA*, *mchF*, *nlpI*, *ompT*, *papC*, *sitA*, *terC*, *traJ*, *traT*, *yehA*, *yehB*, *yehC*, *yehD*, *yghJ*	ColpVC, IncFIB, IncFIC(FII), IncI1-I(Alpha), IncQ1, Col(MG828)	Tn2, ISEc9, MITEEc1, IS629, IS26

**Table 4:** Resistance determinants detected in transconjugants and associated co-transferred phenotypes.

Transconjugant	Donor Strain	Co-Transferred Non-β-Lactam Resistance Phenotype *	Genes Detected in Transconjugant
TC-Ec1	Ec1	TET, CHL	*bla*_CMY-2_, *bla*_TEM-1B_
TC-Ec2	Ec2	SXT, TET, CHL, CIP	*bla*_CTX-M-15_, *bla*_TEM-1B_, *qnrS1*, *sul2*, *dfr*
TC-Ec3	Ec3	SXT, TET, CHL, CIP	*bla*_CTX-M-15_, *bla*_TEM-1B_, *qnrS1*, *sul2*, *dfr*
TC-Ec4	Ec4	TET, CHL	*bla*_CMY-2_, *bla*_TEM-1B_

* Co-transferred phenotype refers to non-β-lactam resistance traits observed in the transconjugants after selection. TC, Transconjugant; TET, tetracycline; CHL, chloramphenicol; SXT, trimethoprim–sulfamethoxazole; CIP, ciprofloxacin.

## Data Availability

Whole-genome sequencing data generated in this study have been deposited in NCBI GenBank/SRA under BioProject number PRJNA1367915 and accession numbers SRR36138959, SRR36138960, SRR36138961, SRR36138962. Other data supporting the findings of this study are available from the corresponding author upon reasonable request.

## Supplementary Materials

The following supporting information can be downloaded at: https://www.mdpi.com/article/10.3390/antibiotics15050449/s1, Table S1: Minimum inhibitory concentrations (MICs, mg/L) of key antibiotics tested against the four MDR *E. coli* isolates.

## Abbreviations

The following abbreviations are used in this manuscript:

AMR	Antimicrobial resistance
BacMet	Bacterial Metal Resistance Genes database
BHI	Brain Heart Infusion
bp	base pairs
BV-BRC	Bacterial and Viral Bioinformatics Resource Center
CARD	Comprehensive Antibiotic Resistance Database
CDS	Coding DNA sequences
cgMLST	Core-genome multilocus sequence typing
CRISPR	Clustered regularly interspaced short palindromic repeats
EUCAST	European Committee on Antimicrobial Susceptibility Testing
ExPEC	Extraintestinal pathogenic *Escherichia coli*
GDL	Deoxycholate lactose agar
MDR	Multidrug resistant/multidrug resistance
MGE	Mobile genetic element(s)
MLST	Multilocus sequence typing
NGS	Next-generation sequencing
PBP3	Penicillin-binding protein 3
PMQR	Plasmid-mediated quinolone resistance
QRDR	Quinolone resistance–determining region
rMLST	Ribosomal multilocus sequence typing
rST	Ribosomal sequence type
ST	Sequence type
TCDB	Transporter Classification Database
VFDB	Virulence Factors of Pathogenic Bacteria database
WGS	Whole-genome sequencing
