# Supplementary material for: NGS-Based Genomic Characterization of ESBL/AmpC-Producing Extraintestinal Pathogenic Escherichia coli from Captive Wildlife in Tunisia
Source: Antibiotics (Basel). 2026 Apr 29;15(5):449. doi: 10.3390/antibiotics15050449 (PMC13203681; doi:10.3390/antibiotics15050449)
Supplement: Supplementary file 1 [file antibiotics-15-00449-s001.zip › antibiotics-4051289-supplementary.pdf]

Supplementary Table S1. Minimum inhibitory concentrations (MICs, mg/L) of key antibiotics tested against the four MDR E. coli isolates

**Antibiotic**	**Method used for MIC**	**EUCAST breakpoints**	**Ec1**	**Ec4**	**Ec2**	**Ec3**
Ampicillin	Broth microdilution	S ≤ 8 — R > 8	128 (R)	256 (R)	32 (R)	128 (R)
Cefotaxime	Broth microdilution	S ≤ 1 — R > 2	>256 (R)	>256 (R)	>256 (R)	>256 (R)
Amikacin	Broth microdilution	S ≤ 8 — R > 8	2 (S)	0.5 (S)	2 (S)	1 (S)
Ciprofloxacin	Broth microdilution	S ≤ 0.25 — R > 0.5	16 (R)	16 (R)	64 (R)	8 (R)
Imipenem	Broth microdilution	S ≤ 2 — R > 4	0.25 (S)	0.5 (S)	0.125 (S)	0.25 (S)
Ertapenem	Broth microdilution	S ≤ 0.5 — R > 0.5	<0.5 (S)	<0.5 (S)	<0.5 (S)	<0.5 (S)
Colistin	Reference broth microdilution	S ≤ 2 — R > 2	1 (S)	0.5 (S)	0.5 (S)	1 (S)
Tigecycline	Gradient diffusion (Etest)	S ≤ 0.5 — R > 0.5	<0.5 (S)	<0.5 (S)	<0.5 (S)	<0.5 (S)

MIC, minimum inhibitory concentration (mg/L); EUCAST, European Committee on Antimicrobial Susceptibility Testing; S, susceptible; R, resistant.
